# Quantum dots implementation as a label for analysis of early stages of EGF receptor endocytosis: a comparative study on cultured cells

**DOI:** 10.18632/oncotarget.6720

**Published:** 2015-12-22

**Authors:** Anna V. Salova, Tatiana N. Belyaeva, Ekaterina A. Leontieva, Maria V. Zlobina, Marianna V. Kharchenko, Elena S. Kornilova

**Affiliations:** ^1^ Institute of Cytology of the Russian Academy of Sciences, St. Petersburg, Russia; ^2^ Peter the Great St. Petersburg Polytechnic University, St. Petersburg, Russia; ^3^ St. Petersburg State University, St. Petersburg, Russia; ^4^ Central European Institute of Technology, Brno, Czech Republic

**Keywords:** EGF receptor, quantum dots, endocytosis, EEA1, HRS

## Abstract

EGF complexed to fluorescent photostable quantum dots by biotin-streptavidin system (bEGF-savQD) is attractive for both the basic research and therapeutic application such as targeted drug delivery in EGF-receptor (EGFR) expressing cancers. However, compared to native EGF, the large size of QD and its quasi-multivalency can have unpredictable effects on EGFR endocytosis changing the internalization portal and/or endosomal processing tightly bound to EGF signaling. We have found that bEGF-savQDs enter HeLa cells via the temperature-dependent clathrin-mediated EGF-receptor-specific pathway characteristic for native EGF. We also found that EGF-to-QD concentration ratios used for the complex preparation and the level of EGF receptor expression affect the number and integral densities of the formed endosomes. So, at EGF-to-QD ratio from 4:1 to 12:1 (at nanomolar bEGF concentrations) on average 100 bright endosomes per HeLa cell were formed 15 min after the complex addition, while 1:1 ratio resulted in formation of very few dim endosomes. However, in A431 cells overexpressing EGFR 1:1 ratio was effective. Using dynamin inhibition and Na-acidic washout we showed that bEGF-savQDs bind surface receptors and enter clathrin-coated pits slower than the same ligands without QD. Yet, the bEGF-savQD demonstrated similar to native EGF and bEGF-savCy3 co-localization dynamics with tethering protein EEA1 and HRS, the key component of sorting ESCRT0 complex. In conclusion, our comparative study reveals that in respect to entrapment into coated pits, endosomal recruitment, endosome fusions, and the initial steps of endosomal maturation, bEGF-savQD behaves like native EGF and QD implementation does not affect these important events.

## INTRODUCTION

During the last decades the progress in cell studies is considerably based on the development of light microscopy techniques and fluorescent markers with advanced optical properties. A new type of fluorophores, non-organic semiconductor nanocrystals known as quantum dots (QDs), became quite popular due to their high quantum yield, excitation in the UV-blue range common to all QDs, and size-tunable narrow emission spectra, combined with high photostability [1, 2]. These properties provide the basis for numerous applications, such as the simultaneous visualization of several intracellular targets excited by a single source, long-lived labeling of the cells of interest, monitoring the behavior of internalized proteins in live cells and control of addressed drug delivery [3–5]. To provide the specificity of QD binding to the cell, a certain ligand or antibody against plasma membrane (PM) proteins should be attached to the particle. Numerous studies concerning cell delivery, toxic effects, intracellular fate of internalized ligands have been conducted using streptavidin-conjugated QDs (savQDs) with biotinylated EGF (bEGF) as the most popular ligand [6–10].

The interest in the EGF-receptor (EGFR) system is explained by its involvement in the regulation of important cell processes such as embryonic development, proliferation, apoptosis, differentiation and cell motility in many cell types [11, 12]. EGF effects are mediated by a highly specific transmembrane receptor possessing intrinsic tyrosine kinase (TK) located in the cytoplasmic domain of the protein. EGF binding to the receptor activates its TK, thus stimulating numerous signaling cascades and, in parallel, internalization of EGF-receptor complexes by clathrin-mediated endocytosis. Internalized EGF-receptor complexes pass through early and late endosomal compartments and are finally delivered to lysosomes for degradation; however, recycling is also possible. The balance between degradation and recycling determines amplitude, duration and endosomal signaling specificity [13–15]. Though the EGF-EGFR system can be considered the most well-studied among different growth factors' families, many important aspects of EGFR functioning are still unclear. Nevertheless, it is established that dysfunction of the EGF-receptor signaling and/or traffic often results in malignization. About one third of human epithelial tumors of the head, neck, lung or colon correlates with receptor overexpression or mutations, so EGFR is deservedly called an “oncoprotein” [16].

Still remaining an important object of basic research, EGFR, at the same time, is a very attractive target for anticancer therapy [17, 18]. Obviously, that a certain application has its own requirements toward a label used which are surprisingly poorly discussed. In basic research the label should not affect the native behavior of the molecule of interest in any way to avoid artifacts. However, when EGF-QD particles with multiple binding sites are used for anticancer drug delivery, the label can alter the course of events but we must know what exactly these changes are. This will help to develop the most efficient design of the labeled particle.

Despite obvious advantages of QDs as a label and delivery vehicle, there are serious doubts in its neutrality. First, the size of a QD bearing several streptavidin tetramers with a molecular mass of about 80 kDa each is much higher than that of native EGF (molecular mass of 6 kDa). According to the manufacturer' estimation the size of savQD is about 15–20 nm [19]. It is well established that all known endocytic portals have specific spatial characteristics, and the clathrin-coated pit (CCP) used by native EGF-receptor complexes for internalization is quite a rigid structure of about 120–150 nm in diameter in human epithelial cells [20, 21]. Second, savQD possesses numerous binding sites for bEGF, making bEGF-savQD complexes quasi-multivalent. However, it is widely recognized now that one EGF molecule binds one receptor molecule [22], but dimerization and possibly tetramerization of 1:1 EGF-receptor complexes is strongly required for internalization and further TK activation. Some lines of evidence suggest an even more complicated mechanism of ligand-receptor complex activation, but this issue is still under debate [9, 23–25].

It can be supposed that the ability of small free ligands and the same ligands bound to comparably large units with numerous spatially different binding sites to form oligomeric receptor complexes with active conformations in the plane of PM can be quite different, and implementation of such a particle can have unpredictable effects on this process – from stabilization of EGFR oligomers to prevention of proper receptor clustering. This may also result in the changing of the way of entry or slowing down the bEGF-savQD internalization as well as disturbance of further early events. The bulk of current knowledge on early endocytic events has come from immunofluorescent imaging of cells stimulated to endocytose native EGF for different time periods and then stained with antibody upon cell fixation [26, 27]. The efficacy of endocytosis depends on the rate and degree of the label accumulation in the coated pits and early endosomes (EE) and is proportional to the integral density and the number of endosomes per cell. Any changes in these parameters will manifest changes in the dynamics of the early stages of endocytosis.

The main aim of this work was to study possible impacts of QD-bound EGF on the early stages of EGF-receptor endocytosis. First, we have evaluated whether the amount of EGF bound to a QD (EGF-to-QD ratio) and the level of EGF receptor expression in the cell affect the number and integral densities of the formed endosomal structures; second, we have analyzed the dynamics of ligand binding, CCP and endosome formation; third, we have estimated the degree and dynamics of co-localization of receptor-containing structures with the markers of consecutive early endocytic events: clathrin (internalization), tether protein EEA1 (early endosome fusions) and ESCRT0 key protein HRS (entering the lysosomal degradative pathway). To estimate the contribution of QD implementation *per se*, we included bEGF labeled with streptavidin-bound Cy3 (bEGF-savCy3) in the analysis. This panel of labels allows to reveal the effect of the size and quasi-multivalency of QD and limitations due to the receptor expression level and capacity of coated pits. To the best of our knowledge, such combined data are still unavailable.

## RESULTS

### bEGF-savQD complexes enter HeLa cells via EGFR-specific pathway

**Figure 1:**
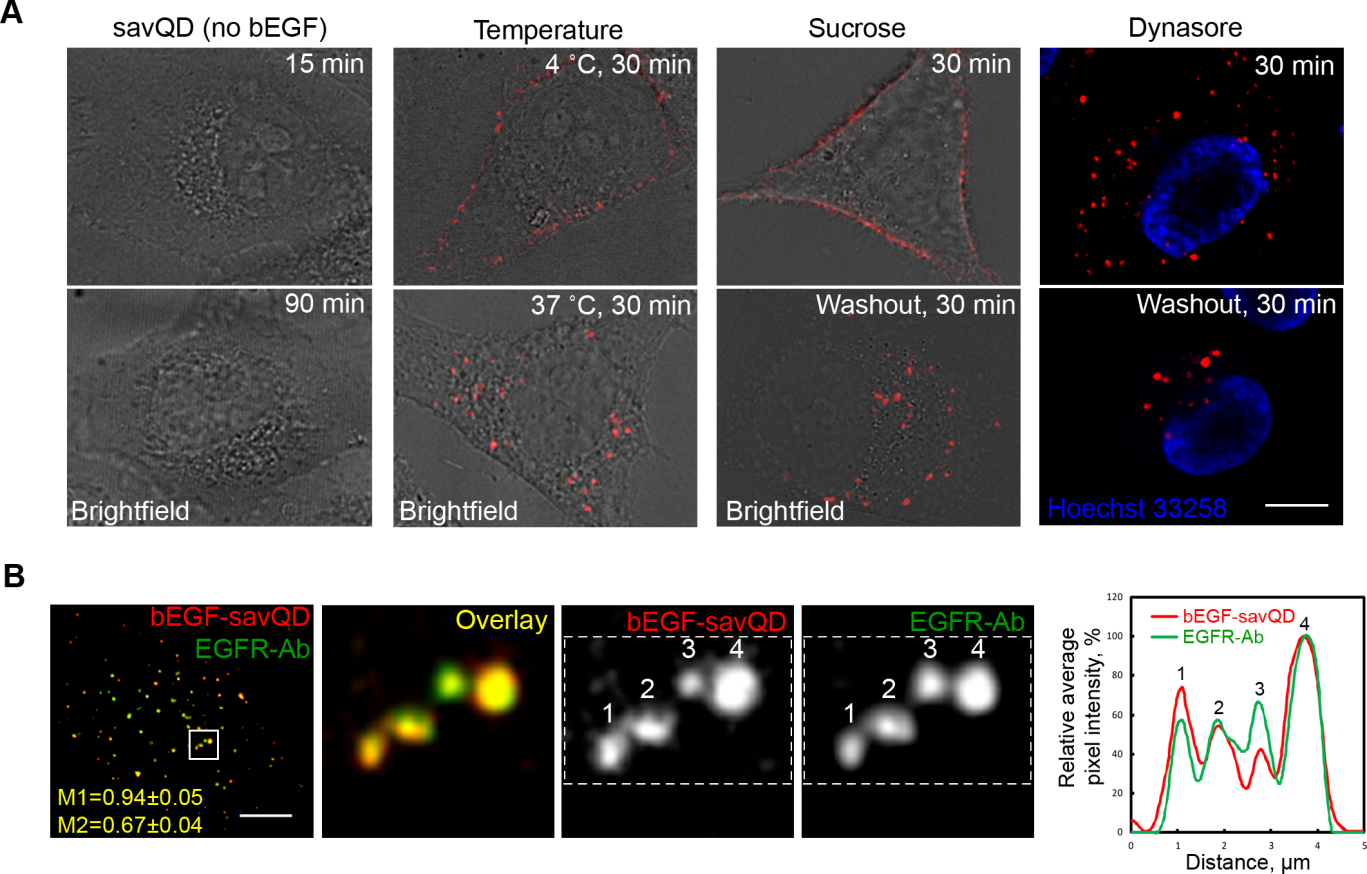
Specificity of bEGF-savQD entry into the cells (**A**) HeLa cells were incubated with savQD (0.5 nM) or bEGF-savQD (2:0.5 nM) for the indicated periods under different conditions. Live cells were analyzed. The images are presented as single sections from the region of maximal cell spreading. (**B**) HeLa cells were incubated with bEGF-savQD (2:0.5 nM) for 15 min at 37°C, fixed and immunostained with anti-EGFR antibody (Alexa 488) before confocal microscopy. The image is the projection of Z-series onto a single image using a max intensity method (ImageJ). Manders' coefficients were 0.94 ± 0.05 (M1, bEGF-savQD overlapping EGFR) and 0.67 ± 0.04 (M2, EGFR overlapping bEGF-savQD). Enlarged views (8.8 ×) of boxed region are shown in the next images. The plot profile of the allocated area including 4 endosomes shows the intensities of bEGF-savQD and EGFR-Ab in each endosome. Each image is representative of at least three independent experiments. Scale bars: 10 μm.

To confirm that EGF complexed to QDs follows exactly the same pathway as native EGF at the very early endocytic stages, we tested several key characteristics of the ligand interaction with HeLa cells. First, it was shown that savQDs failed to bind the cell membrane in the absence of bEGF, either after 15 min or 90 min of incubation with the cells (Figure 1A, left column). Second, it is known that EGF binds its receptor at the plasma membrane at 4°C, but internalization requires higher temperatures, with an optimum at 37°C [13]. Indeed, the preformed bEGF-savQD complexes were localized only at PM at 4°C, but were clearly detected intracellularly after warming up to 37°C (Figure 1A, second column). Third, the clathrin-dependent mechanism of internalization was proven in two ways: by the destruction of clathrin lattice at high sucrose concentrations and the specific inhibition of atypical GTPase dynamin, a key component of clathrin-coated pit constriction, by dynasore (Figure 1A). The images presented in the two right columns demonstrate that 30 min incubation with both drugs results in exclusively membrane localization of the complexes. The washout of sucrose or dynasore followed by 30 min incubation in drug-free culture medium results in the formation of QD-labeled structures in the juxtanuclear region of the cell, which is typical for endocytosis of native EGF. The analysis of cells, allowed to internalize bEGF-savQDs for 15 min and then stained with an antibody against EGF receptor (Figure 1B), showed that practically all bEGF-savQDs-positive vesicles were co-localized with EGF receptor (M1 – 0.94 ± 0.05). Co-localization of EGFR-containing structures with QD was insignificantly lower (M2 – 0.67 ± 0.04), which may indicate a portion of uninternalized unbound receptors under the conditions used or some endosomes loaded by QD-unbound bEGF. However, in the cell presented in Figure 1B as well as in all 60 analyzed cells such endosomes were very few. Thus, we can conclude that the preformed bEGF-savQD complexes bind HeLa cells by the EGF-EGFR-specific mechanism and are internalized by the temperature-dependent, clathrin-mediated pathway characteristic for native EGF.

Importantly, the dual detection of both the QD-labeled EGF and EGFR in fixed cells also shows that the bEGF-savQD:EGFR ratio varies from endosome to endosome (Figure 1B): the plot profile of the selected area including 4 endosomes indicates that in vesicle №3, the mean intensity of EGFR staining is higher than that of QD, while the opposite is observed for vesicle №1. Most probably, this reflects the non-uniform mode of bEGF binding to savQD, and the abovementioned stoichiometric problems in EGF/EGFR dimer formations, possibly associated with the relatively large size of savQD particles.

**Figure 2:**
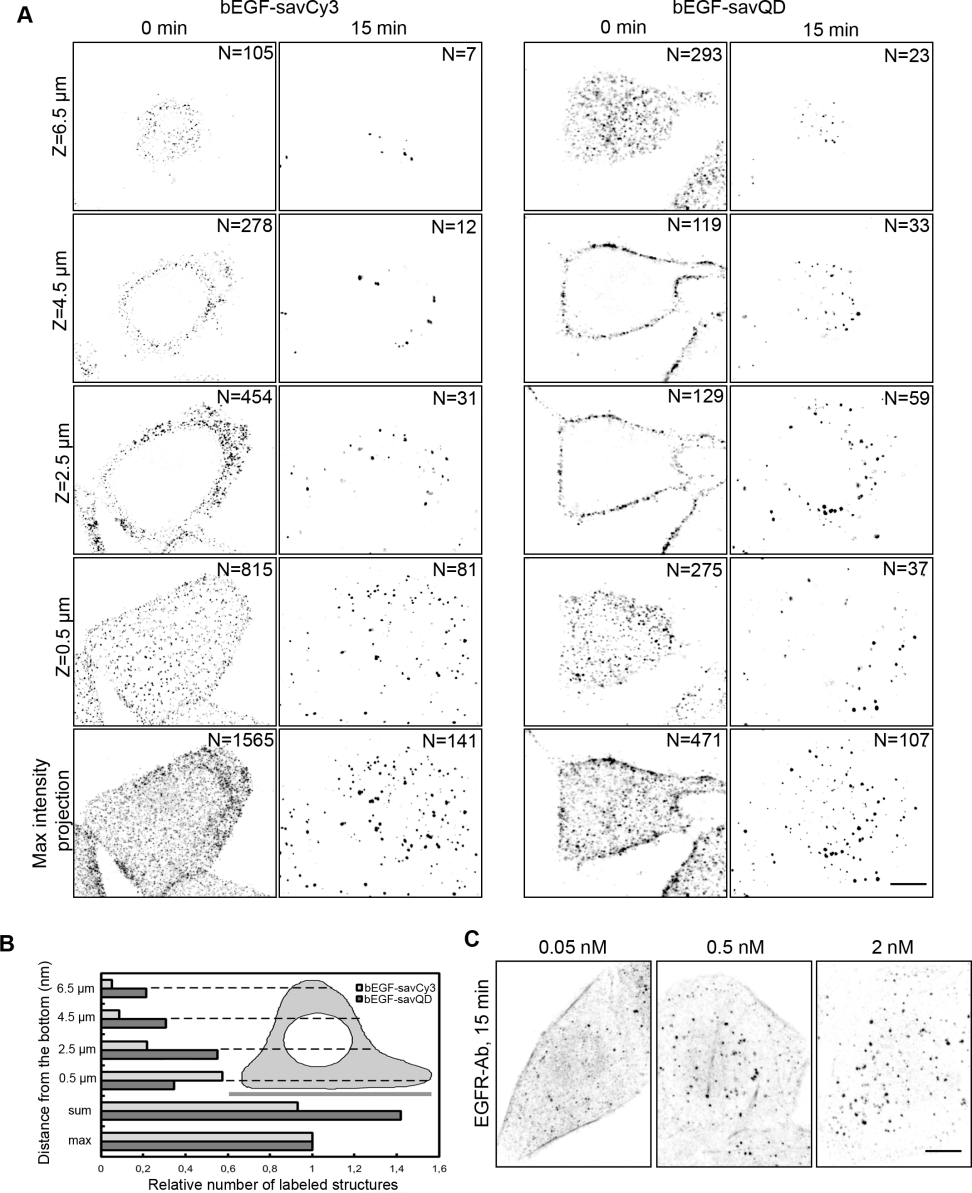
Investigation of bEGF-savCy3 and bEGF-savQD distribution on PM and after internalization into the cells (**A**) HeLa cells were incubated with bEGF-savCy3 (2 nM) or bEGF-savQD (2:0.5 nM) using prebinding (0 min) at 4°C and endocytosis stimulation by shifting the cells to 37°C medium (15 min); then the cells were fixed before confocal microscopy. Z-series optical sections were taken at 0.5-μm intervals (14–16 sections). The images represent 4 single sections from Z-stacks and a projection of Z-stack onto a single image obtained by max intensity method (ImageJ). The number of labeled structures is indicated for each section and max intensity projection. (**B**) Z-stack analysis of relative number of labeled structures upon 15 min of endocytosis from (A) is represented. (**C**) HeLa cells were incubated with indicated concentrations of EGF using pulse-chase (15 min) protocol, fixed and immunostained with anti- EGFR antibody (Alexa 488) before confocal microscopy. The images represent the projection of Z-stacks onto a single image. These data are typical of at least three independent experiments. Scale bars: 10 μm.

To find out whether the QD size affects the surface receptor binding and internalization efficacy, we have compared the distribution of bEGF-savQD and bEGF-savCy3 on PM after prebinding at 4°C and after 15 min of endocytosis stimulated by shifting the cells to 37°C medium. Note that unlike relatively small savCy3, savQD possesses 5–10 streptavidins according to manufacturer's estimations. We have found that the two prebound markers were detected in clusters distributed relatively uniformly onto membrane domains oriented towards the culture medium (Figure 2A), but the smaller ligand bEGF-savCy3 more extensively labeled the cell surface oriented towards the coverslip (see Z-section at 0.5 μm), while bEGF-savQDs were mostly concentrated “apically” (at 6.5 μm) and close to the cell edge (at 2.5 and 4.5 μm). Quantitative estimations of the prebound label gave a higher number of separate structures for savCy3 than for savQD (maximal intensity projection protocol shows 1565 separate structures versus 471, respectively). This may reflect the formation of small but numerous clusters by savCy3 and larger but fewer clusters by savQD.

Despite this difference, in 15 min of endocytosis, a similar number of EGF-positive endosomes was detected for both labels (141 and 107, respectively). Interestingly, at 15 min, the simple summation of numbers of the detected endosomes in all optical slices produced higher results for QD- compared to Cy3-labeled structures (Figure 2B), while the number estimation according to the protocol of maximal intensity projections gave lower values for QDs. This indicates that QD is brighter and the same structure can be seen in more than one neighboring slice made with 2 μm steps along the Z axis, even though the physical size of an endosome is barely more than 1 μm. Importantly, endosome formation in the case of bEGF-savCy3 occurs mostly from basal and lower parts of the lateral domains of PM, whereas bEGF-savQDs preferred conventionally apical PM domain, which corresponded to the initial distribution of the labels (Figure 2A and scheme B).

Probing EGF receptor distribution with an antibody shows more diffuse pattern (Figure 2C), and at low EGF concentrations (0.05 nM) strong signal from the PM makes it difficult to reliably detect endosomes. Even at 0.5 nM EGF, there is still a significant portion of receptors remaining at the PM, producing high background. Therefore, at such non-saturating EGF concentrations unligated receptors are localized out of specific domains that are able to concentrate them, like CCPs. Only at 2 nM of EGF on HeLa cells all surface receptors become internalized into endosomes, thus abolishing the background signal. It should also be noted that detection of PM receptors by externally applied labeled ligands makes it possible to follow the fate of only ligated receptors in comparison with the traditional determination using antibody staining of the total receptor population. However, in the receptor overexpressing cells like A431, even saturating EGF concentrations (about 20–30 nM) fail to promote redistribution of all receptors to endosomes, thus producing a high background signal (data not shown).

### The efficacy of internalization depends on bEGF to savQD ratio and EGF receptor expression level

**Figure 3:**
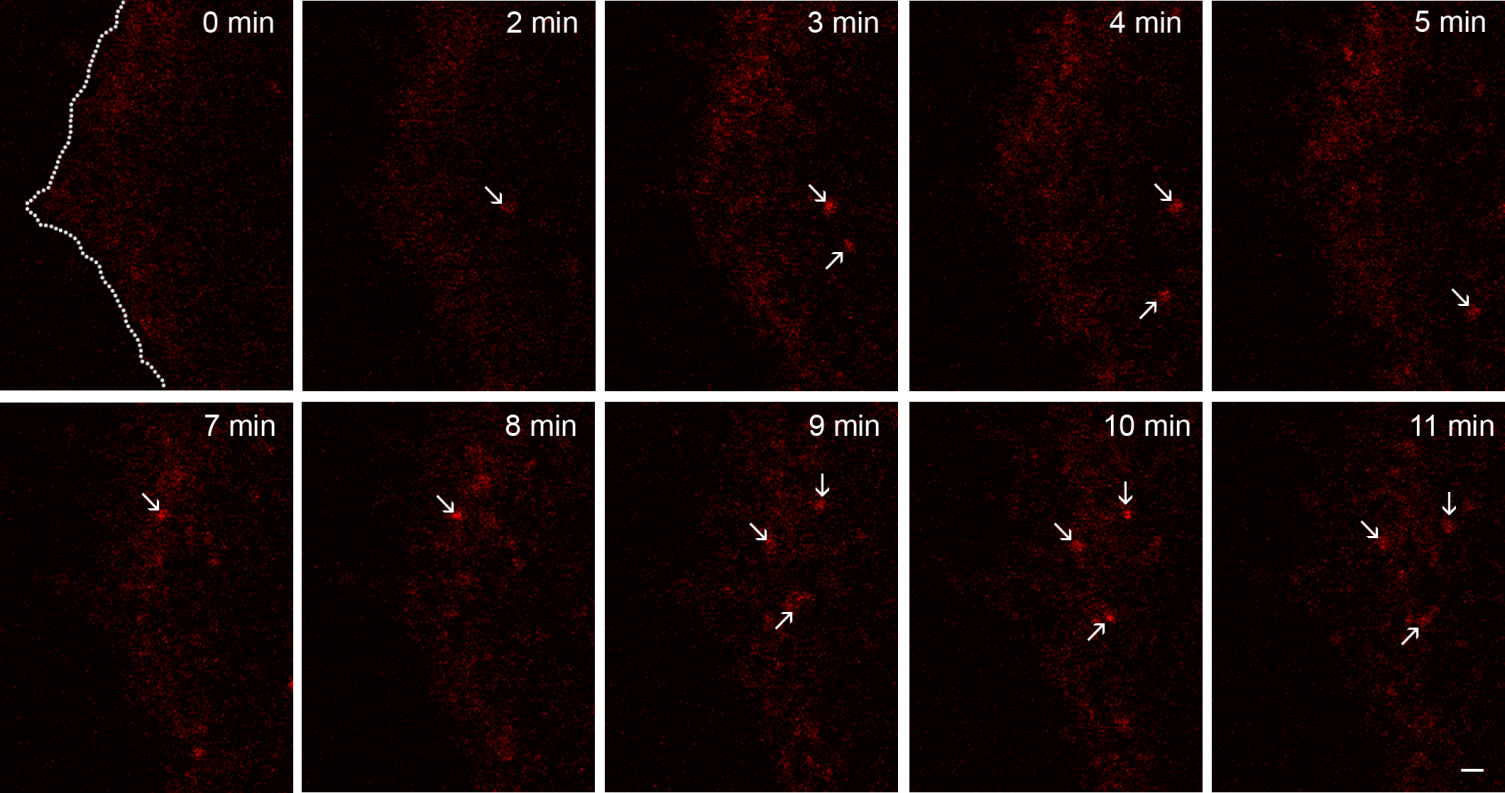
Early stages of endosome formation Frames from a representative time-lapse video (see Supplementary Video) illustrating the formation of endosomes after the addition of bEGF-savQD (2:0.5 nM) complexes to PAE A11 cells (11 min duration). Note the highly dynamic meshwork of fluorescent dots appearing and disappearing in areas close to the cell edge marked in the first frame by a dotted line and emerging bright structures indicated by arrows. Scale bar: 1 μm.

When bEGF-savQD complexes were added to the cells according to the pulse-chase protocol (which is more physiological than prebinding at 4°C followed by warming the cells up to 37°C), several different processes go in parallel smoothing the outcome: association/dissociation of EGF and its receptor at non-steady state conditions, dimerization/clasterization of already bound complexes, their recruitment into clathrin-coated pits, assembly of clathrin-coated vesicles, pinching them off into the cytoplasm, possibly fusions of just formed endosomes and their recycling. To visualize these early events in live cells we have used PAE A11 epithelial line for two reasons. First, these cells express a high number of EGF receptors fused to GFP [28], which allows to detect simultaneously EGF-receptor positive structures and QDs. Second, PAE A11 cells are very flat (2–3 μm) at the periphery regions, so the formed CCPs/endosomes practically did not come out of the focal plane that is important for estimating the behavior of EGF-receptor complexes. Due to light microscopy resolution limitations and the non-planar profile of the plasma membrane, one cannot differentiate between QD particles: (i) localized in close neighborhood with PM; (ii) already attached to PM; or (iii) just false background signals. Supplementary Figure 1A demonstrates that bEGF-savQD in PAE A11 cells is localized only in receptor-positive structures 15 min after endocytosis, which is proved by high Manders' co-localization coefficient (M1) and indicates the specificity of bEGF-savQD internalization. However, live cell imaging study (Figure 3 presents a series of frames from a corresponding Supplementary Video) shows the highly dynamic meshwork of dimmed fluorescent dots appearing and disappearing in the area close to the cell edge during the first 5–10 min after the addition of bEGF-savQD to the cells. Despite the dotted pattern of QD signal, the data from the green channel registering the EGFR-GFP signal demonstrated a rather uniform distribution of the receptor in the membrane (see Supplementary Video). Additionally, it can be seen that the high density of PM-localized EGF receptors does not allow to detect structures with low GFP signal concentration. Both the red and green structures of such low intensity are excluded at background thresholds used for particle analysis by standard software programs like ImageJ. However, only after 5–15 min relatively bright stable QD-positive spots behaving as single entities can be reliably detected. They could be clathrin-coated pits (CCPs) as well as primary endosomes, but they undoubtedly contain a highly concentrated cargo. Importantly, the video clearly demonstrated that QD signals are stable during all the experiment, while the GFP signal undergoes very fast bleaching, and could be detected only in QD-positive structures thus proving EGFR concentration there. Thus, to reliably estimate the characteristics of QD-positive structures using imaging analysis programs, the 15-min time point was chosen for the majority of further experiments. It must be underlined that according to the numerous studies formation of an endosome takes about 2 min [29]. A delay in appearance of bright structures indicates that the process of bEGF-savQD binding to the receptor and formation of stable complexes takes a significant amount of time. A high level of ligand concentration in the structures recorded at 15 min together with the earlier data on endocytosis dynamics [29, 30] indicates that the majority of QD-containing structures are endosomes.

The movie presented illustrates fast photobleaching of GFP fluorescence. Comparing QD-labeled endosomes with those stained with another fluorophores used in our study, Cy3 and Alexa488, we also detected a significant decrease in the intensities of endosomes in fixed HeLa cells only for Cy3 and Alexa488 (Supplementary Figure 2). The degree of photobleaching was proportional to the surface power density. Importantly, the fluctuations of intensity of the indicated QD-labeled endosomes with time have a character which is often interpreted as photoblinking and gives the grounds to claim that the fluorescence is produced by a single QD. In this study we demonstrate that such a conclusion may be incorrect because: (i) not only one but several QDs can cause such flickering; and (ii) the same pattern is demonstrated by bEGF-savCy3 which does not possess any quantum properties. Besides, as was discussed above, Figure 3 (see also Supplementary Video) has proved that single bEGF-savQD particles initially interacting with surface receptors are hardly seen before they become concentrated in endosomes.

**Figure 4:**
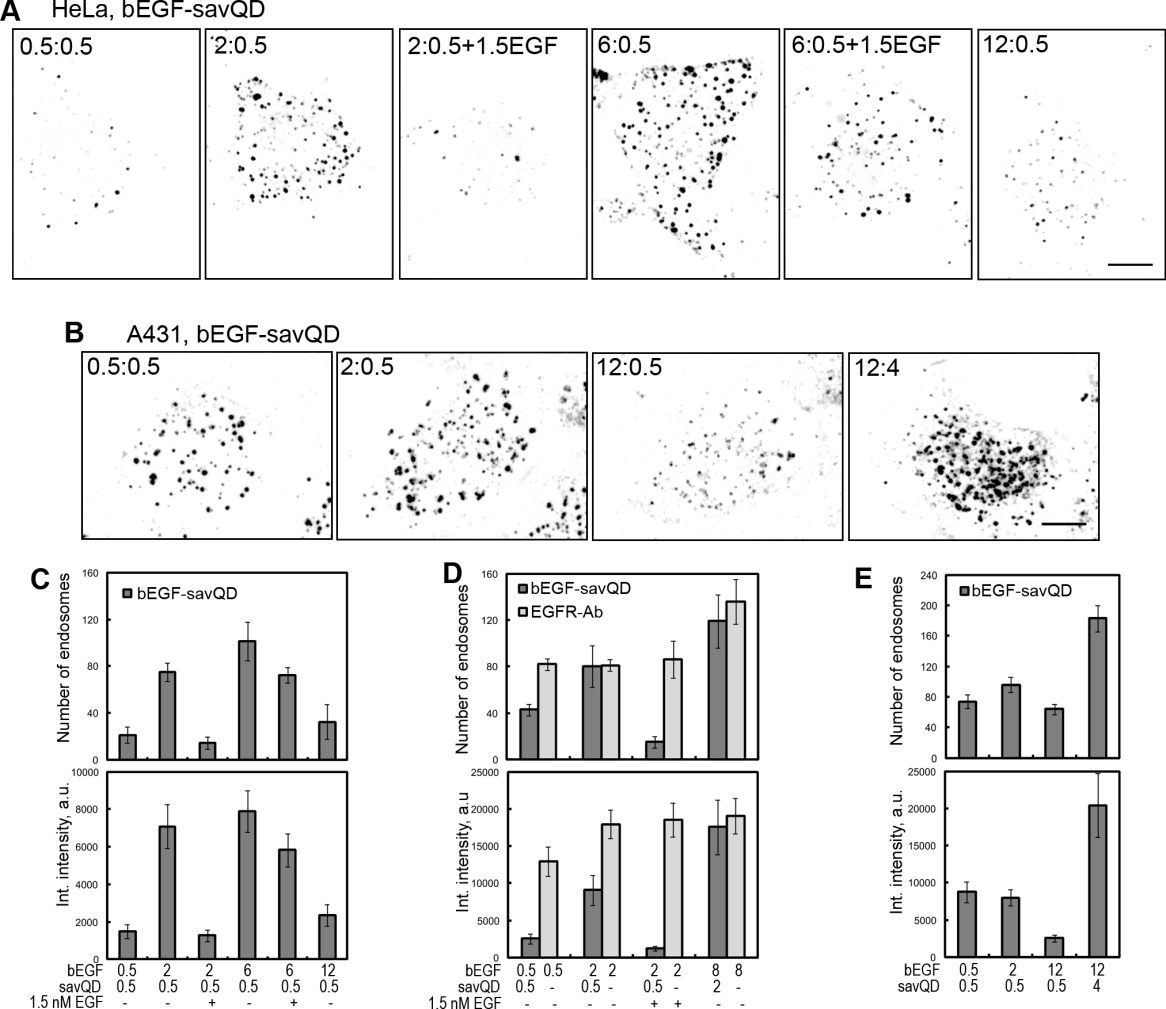
The influence of variable ratios of bEGF to savQD concentrations on the formation of CCP/EE in the cells (**A**) HeLa cells were incubated with bEGF-savQD (the concentration of bEGF varied from 0.5 to 12 nM with the addition of 1.5 nM free EGF in some cases and the concentration of savQD was 0.5 nM) using pulse-chase (15 min) protocol and fixed before confocal microscopy. (**B**) A431 cells were incubated with the indicated concentration ratios of bEGF to savQD using the pulse-chase (15 min) protocol and fixed before confocal microscopy. (**C**) The number of endosomes and their integrated intensity (for HeLa cells from the experiment described in (A) were calculated from max intensity projections of the cells for every bEGF to savQD ratio using ImageJ. (**D**) HeLa cells were incubated with bEGF-savQD (0.5:0.5, 2:0.5 or 8:2 nM) or bEGF (0.5, 2 or 8 nM) using the pulse-chase (15 min) protocol; in some cases, 1.5 nM of free EGF was added, then the cells were fixed and in the case of bEGF immunostained with anti-EGFR antibody (Alexa 488) before confocal microscopy (images are not shown). The number of endosomes and their integrated intensity were calculated from the max intensity projections of the cells for every concentration using ImageJ. (**E**) The number of endosomes and their integrated intensity (for A431 cells from experiment described in (B) were calculated from max intensity projections of the cells for every bEGF to savQD ratio using ImageJ. Each image is representative of at least three independent experiments. The inverted images are projections of Z-series onto single images obtained by max intensity method (ImageJ). Scale bars: 10 μm. Data presented as the mean ± 95% confidence interval of three independent experiments.

As mentioned above, bEGF-savQD unlike native EGF is a quasi-multivalent ligand for EGF receptors: a QD particle bears 5–10 streptavidins each of which has 4 binding sites for biotin; so there could be spatial problems hampering efficient dimer formation. Obviously, the efficacy of dimer formation should grow with the increase in bEGF to savQD ratio. To test this assumption, complexes preformed at variable ratios of bEGF to QD concentrations were used to evaluate the number and integral density of CCP/EE 15 min after endocytosis stimulation (Figure 4). Starting with low concentrations of bEGF far from receptor saturation in HeLa cells, we found that 0.5 nM bEGF added to 0.5 nM of QDs (1:1 ratio) was not effective for CCP/EE formation: the number and brightness of QD-positive structures were very low, which is clearly seen in the representative image and corresponding quantitative evaluation (Figure 4A and 4C). However, free EGF at 0.5 nM was efficiently internalized (Figure 4D, compare two left columns). The increase in bEGF concentration up to 2 nM (4:1 ratio) resulted in a drastic increase in the number of QD-positive structures and their mean integral intensity, which is proportional to the fluorophore concentration in the structure. However, bEGF at a concentration of 6 nM (12:1 ratio) insignificantly increased the number of QD-positive structures which, as in the previous case, had a high integral density. A further increase in bEGF concentration (up to 24:1 ratio) led to a pronounced drop in both the number and brightness of CCP/EE, which is most probably due to the exceeding the limit of binding sites on savQDs and thus appearing of unbound bEGF in the incubation medium. This free bEGF molecules displace QD-complexes from the receptors. Our data suggest that the majority of savQD used in this series was conjugated to significantly fewer than 6 streptavidin tetramers each or that the attachment of bEGF to each binding site of streptavidin tetramer is not a rule even in the situation of the bEGF excess.

This assumption is in agreement with the data on the strong competitive effect of free EGF versus QD-labeled ligand. EGF added in 1.5 nM concentration to the cells together with bEGF-savQD (preformed by mixing 2 and 0.5 nM, respectively) significantly decreased the ability of QD-labeled EGF to bind the cells and form CCP/EE (Figure 4A and 4C). The decrease was seen even at a bEGF to savQD ratio of 6:0.5 (Figure 4A and 4C). Importantly, when the cells treated with 2 nM bEGF together with 1.5 nM EGF were fixed and stained with antibodies against EGFR, the total number of detected receptor-positive structures was found to be the same as that with 2 nM bEGF only (Figure 4D, light grey columns). This indicates that native EGF has a higher affinity for receptors compared to its complex with QD or that the concentration of bEGF-savQDs capable of forming complexes with the receptor is significantly lower than the designated one. Our data also show that free EGF behaves as a competitor rather than a promoter of the formation of dimers/oligomers with bEGF-savQD during receptor binding. The difference in affinity is not due to biotinylation as EGF and bEGF behave similarly (not shown).

In the next series, we raised both bEGF and savQD concentrations 4-fold, keeping an effective ratio of 4:1 (8 nM bEGF to 2 nM savQD). The total number of detected receptor-positive structures containing bEGF-savQD and EGFR-Ab was found to be the same and has grown by 30% (Figure 4D). However, in the case of free bEGF the mean integral density of CCP/EE was found to be maximal even at 2 nM (Figure 4D, light grey columns), while the mean integral density of QD-labeled structures reached the maximum only at 8 nM (ratio 4:1) (Figure 4D, dark grey columns). Taking into account that 2 nM of EGF is lower than the saturating concentration, while 8 nM of EGF is oversaturating for HeLa cells possessing about 3–4 × 10^5^ receptors per cell, it is possible to suggest that native EGF fills CCPs to the maximal concentration faster than in the case of the QD-labeled ligand. Although the design of the experiment does not provide the possibility to distinguish between CCPs and endosomes, a similar number of structures at 15 min of endocytosis suggests that the number of areas allowed for CCP formation is limited.

Another parameter that can influence the internalization efficacy is the level of receptor expression. If HeLa cells have about 3–4 × 10^5^ receptor molecules on PM which become saturated at EGF concentration of about 4 nM, the human epidermoid carcinoma cell line A431 possesses about 2–3 × 10^6^ EGFR per cell which become saturated at about 25–30 nM of EGF. Due to this property A431 cells have been the most popular model for EGFR studies since 1980th. We have found that bEGF-savQD at the concentration used enter A431 cells by the EGFR-specific pathway (Supplementary Figure 1B). However, a high receptor density in these cells results in the significant level of internalization of unoccupied non-activated receptors [31]. Indeed, we have found that bEGF-savQD at internalization ratio ineffective for HeLa cells (1:1) bound and entered A431 cells with efficacy compared with the 4:1 complex (Figure 4B and 4E). Changing the ratio to 24:1 (12 nM bEGF to 0.5 nM savQD) resulted in the same effect as for HeLa cells due to free bEGF excess, but an 8-fold increase in QD concentration (12 nM bEGF to 4 nM savQD) produced a 2-fold increase in the number of CCPs/EEs and more than 2-fold growth of the QD concentration per labeled structure (Figure 4E). Therefore, it can be concluded that in the cells overexpressing EGFR, (i) the maximal number of CCPs/EEs that can be formed is higher than in non-overexpressing cells, but this number is not linearly proportional to the total receptor amount, and (ii) the most internalization efficient concentrations of EGF and QD can be higher than for cells with a relatively low receptor number. However, the rule of “internalization effective ratio” of bEGF and savQD also works.

### Differentiation of PM-bound and internalized bEGF-savCy3 and bEGF-savQD

In the protocol used above, we did not differentiate between bEGF-savQD complexes clustered at PM, accumulated in CCPs and those internalized into endosomes during a 15 min pulse. To investigate the influence of QDs on these consecutive stages in more detail, we compared parameters of the structures labeled by bEGF-savCy3 or bEGF-savQDs. To distinguish between PM-bound and internalized Cy3 and QD we used dynasore that inhibits activity of dynamin [32] – the key protein involved in the constriction of CCP and pinching off of CCP-derived vesicles into the cytoplasm. Inhibition of clathrin-dependent endocytosis was combined with the standard procedure of EGF washout by acetic buffer with a pH of 4.0, which was shown to effectively dissociate EGF-receptor complexes [26].

**Figure 5:**
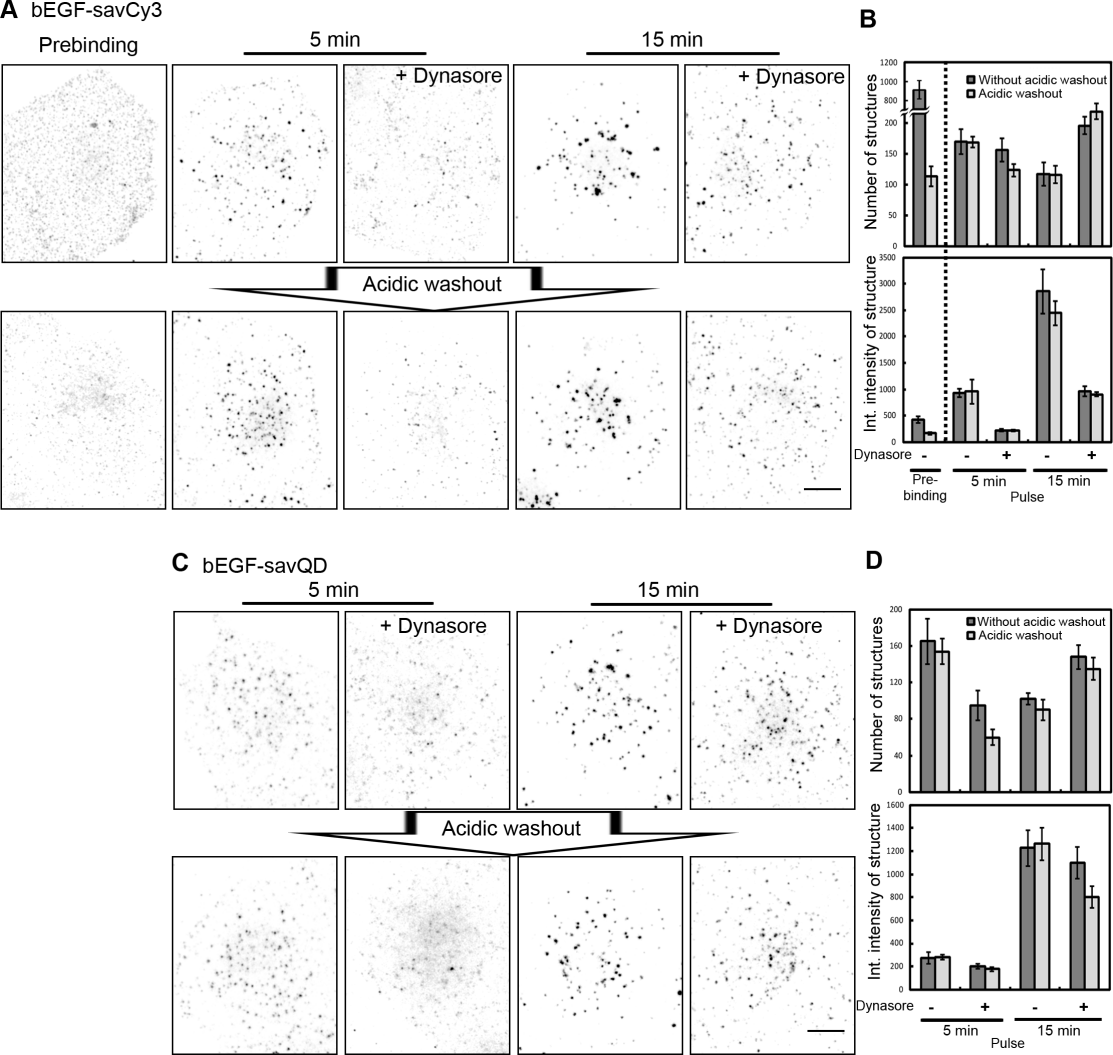
Differentiation of PM-bound and internalized bEGF-savCy3 and bEGF-savQD (**A**) Control or dynasore-treated (80 μM) HeLa cells were incubated with bEGF-savCy3 (2 nM) using prebinding (0 min) or pulse-chase (5 and 15 min) protocols, followed by fixation or acidic washout with pH 4.0 buffer (0.2 M acetic acid/0.5 M NaCl) and fixation. (**B**) The number of structures and their integrated intensity for the cells (from experiment described in A) without or after acidic washout were calculated from max intensity projections for every condition using ImageJ. (**C**) Control or dynasore-treated (80 μM) HeLa cells were incubated with bEGF-savQD (2:0.5 nM) using pulse-chase (5 and 15 min) protocol, followed by fixation or by acidic washout with pH 4.0 buffer and fixation. (**D**) The number of structures and their integrated intensity for the cells (from the experiment described in C) without or after acidic washout were calculated from max intensity projections for every condition using ImageJ. Each image is representative of at least three independent experiments. The inverted images are projections of Z-series onto single images obtained by max intensity method (ImageJ). Scale bars: 10 μm. Data presented as the mean ± 95% confidence interval of three independent experiments.

Prebinding of bEGF-savCy3 at 4°C (when internalization is blocked) to HeLa cells resulted in the formation of EGF-receptor complexes visualized as numerous randomly distributed small spots with low integral fluorescence, reflecting a low degree of label concentration (Figure 5A, Prebinding). Acidic washout of these cells led to a dramatic decrease in both the number and brightness of these spots demonstrating the easy availability of the label for outside treatment. Quantitative image analysis showed that the initial number of Cy3-positive structures dropped about 10-fold and the integral density of the structures became close to background values (Figure 5B, Prebinding). These data favor bEGF-savCy3-receptor complex localization at the PM in small clusters, mainly located outside CCPs. Stimulation of bEGF-savCy3 endocytosis in such control cells for 5 and 15 min of 37°C pulse resulted in an increase in size and brightness of the labeled structures and decrease in their number (Figure 5A and 5B, Pulse). They became practically unavailable for acetic buffer washout; this indicates that these labeled structures are endosomes which underwent fusions.

It could be expected that inhibition of dynamin preventing CCP constriction would result in an increase in both CCP number and availability of yet uninternalized bEGF-savCy3 to acidic washout. However, compared to control cells, dynasore produced only 15–20% increase in CCP number 15 min after endocytosis stimulation (Figure 5B). At the same time, dynasore practically abolished ligand concentrating in CCPs after 5 min pulse but failed to prevent its accumulation by 15 min; however, the integral intensity of CCP reached only about one third of this value in control cells (Figure 5B). Thus, dynasore significantly slowed down the rate of bEGF-savCy3 accumulation in CCPs compared to control. Additionally, the labeled structures were more randomly distributed on the cell surface (Figure 5A). This difference may be explained by internalization of a portion of CCPs and further concentration of the labeled EGF-receptor complexes due to endosome-to-endosome fusions in control versus dynasore-treated cells, when the ligand was located only on PM both non-associated and associated with CCPs. To prove this assumption, we compared the effect of ubiquitously used acidic buffer washout procedure on the control and dynasore-treated cells. Surprisingly, no reliable difference was found except for the label distribution in dynasore-treated cells: acetic washout removed the most dimmed spots localized primarily at the edge of cells, so that their contribution to mean integral density value was negligible (Figure 5A and 5B). Besides, binarization of the images as a necessary step of particle analysis resulted in the apparent disappearance of the structures having small size and low brightness.

Interestingly, quasi-multivalent ligand of bigger size, bEGF-savQD behaved similarly to smaller bEGF-savCy3 in control and dynasore-treated cells (Figure 5C and 5D). However, in contrast to bEGF-savCy3, in the presence of dynasore acetic washout removed 20–30% of the bound bEGF-savQDs even at 15 min (Figure 5D). It means that QD-labeled ligand enters CCPs slower than bEGF-savCy3 and more bEGF-savQD-receptor complexes stay out of CCPs even at 15 min. The comparison of brightness of QD-positive structures in control and dynasore-treated cells at that time point (Figure 5D) also confirms that accumulation of the label in CCPs goes slower than of the ligand without QD.

### bEGF-savCy3 and bEGF-savQD co-localization with early endosomal markers

To further investigate whether the structures we register are still clathrin-coated pits or already early endosomes, we analyzed their co-localization with clathrin, early endosomal autoantigen EEA1 and the key component of the first sorting ESCRT0 complex, HRS, which are believed to mark consecutive steps of EGF receptor endocytosis.

**Figure 6:**
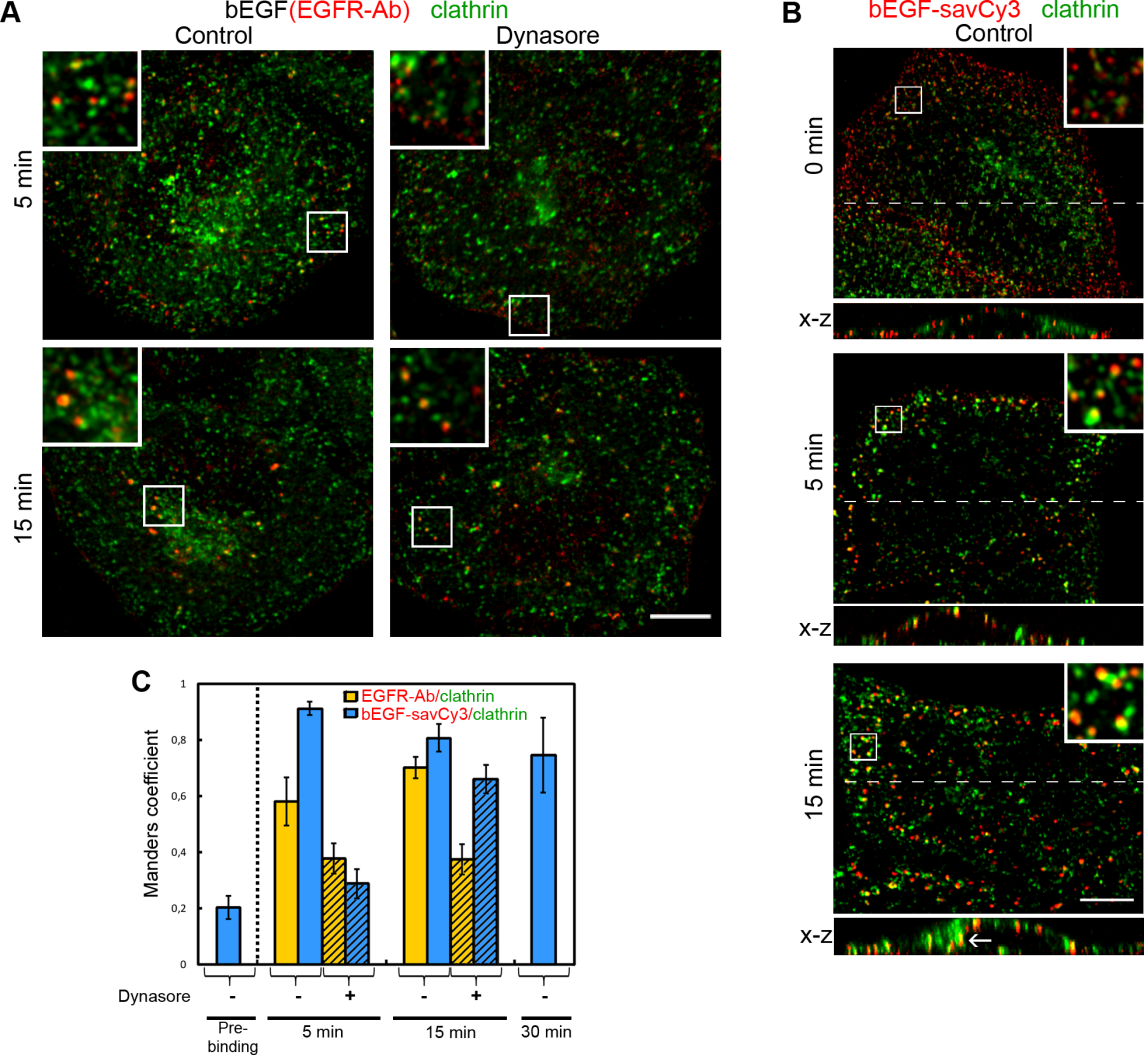
Immuno-co-localization of EGFR or bEGF-savCy3 with clathrin (**A**) Control or dynasore-treated (80 μM) HeLa cells were incubated with bEGF (2 nM) using pulse-chase (5 and 15 min) protocol, fixed and immunostained with anti-EGFR (Alexa 568) and anti-clathrin heavy chain (Alexa 488) antibodies before confocal microscopy. Insets represent enlarged views (2.6 ×) of boxed region. (**B**) HeLa cells were incubated with 2 nM bEGF-savCy3 (red) using prebinding (0 min) or pulse-chase (5 and 15 min) protocols, fixed and immunostained with anti-clathrin heavy chain antibody (Alexa 488) before confocal microscopy. Each image is also represented as sections in the *x*-*z* plane taken from the region indicated by a dotted line. Insets represent enlarged views (3 ×) of boxed region. (**C**) Co-localizations between EGFR-Ab or bEGF-savCy3 and clathrin in control (open columns) and dynasore-treated (shaded columns) cells were quantified using the Manders' coefficient (M1: red pixels overlapping green). Data presented as the mean ± 95% confidence interval of three independent experiments. Each image is representative of at least three independent experiments. The images are single sections from the region of maximal cell spreading. Scale bars: 10 μm.

The probing of EGFR localization with specific antibody in cells stimulated with bEGF (2 nM) for 5 min and 15 min pulses (Figure 6A) shows two main types of EGF receptor-containing structures: (1) peripherally located dot-like receptor positive structures many but not all of which are co-localized with clathrin at 5 min, and (2) large vesicles or structures of more complicated shape, located in juxtanuclear areas and containing high level of clathrin at 15 min. Our estimations of two markers' co-localization show that about 60–70% of pixels positive for total EGFR also contain clathrin signal (Figure 6C). However, only two thirds of this co-localization can be attributed to CCPs, because dynasore treatment results in a 1/3 to 1/2 decrease (by 5 and 15 min, correspondingly) in M1 value, thus indicating that a significant portion of EGF-receptor complexes rapidly becomes endocytosed into endosomes associated with flat clathrin coats reported to participate in the early steps of cargo concentration and sorting to degradative pathway [32, 33]. Enlargement of these structures and their translocation to the juxtanuclear area with time (Figure 6A) support this view. It must be mentioned that in the case of Ab staining of fixed cells, co-localization degree can be underestimated due to detection of the total but not only activated receptor pool.

Indeed, in the case of bEGF-savCy3, which labels only activated receptors, co-localization (M1) was very high at 5 min of pulse and only slightly decreased later on, and was higher than in the case of bEGF alone (Figure 6B and 6C). However, dynasore treatment resulted in a drastic decrease in label co-localization with clathrin (Figure 6C, images are not shown) suggesting that in control, about two thirds of bEGF-savCy3 bound to activated receptor were already internalized at 5 min pulse. In control cells, a number of peripheral bEGF-savCy3 vesicles can be seen that were negative for clathrin staining, as was the case for bEGF alone (Figure 6A and 6B, 5 min). Analysis of *x-z* sections demonstrated that such vesicles localize very close to PM. More probably, these structures present already pinched off uncoated primary endocytic vesicles that are not yet captured by microtubules and only at 15 min we can clearly see intracellular localization of enlarged endosomes, even at the optical resolution limitations of *x-z* projection (Figure 6B, bottom image). This view is supported by our data on the insensitivity of the label to acidic washout at 5 min (Figure 5A and 5B). Importantly, bEGF-savCy3 prebound to PM at 4°C (which was sensitive to acidic washout) demonstrated a very low level of co-localization with clathrin (Figure 6B, upper image). Thus, in control cells bEGF-savCy3 rapidly undergoes internalization into small primary endosomes that later on fuse and become associated with clathrin sorting platforms, so that high co-localization at 15 min and later on is mostly due to intracellularly located processes. On the contrary, longer incubation of dynasore-treated cells with bEGF-savCy3 results in stabilization of activated ligand-receptor complexes in PM-located CCPs (Figure 6C).

Although these results are in line with our abovementioned data, it should be concluded that clathrin is not a very good marker for discrimination between PM-bound and internalized ligands, not only because it is located on numerous different structures except CCPs and endosomes [34–36]. Indeed, the high level of cytoplasmically located clathrin can produce some false signals leading to incorrect estimations of the degree of co-localization.

**Figure 7:**
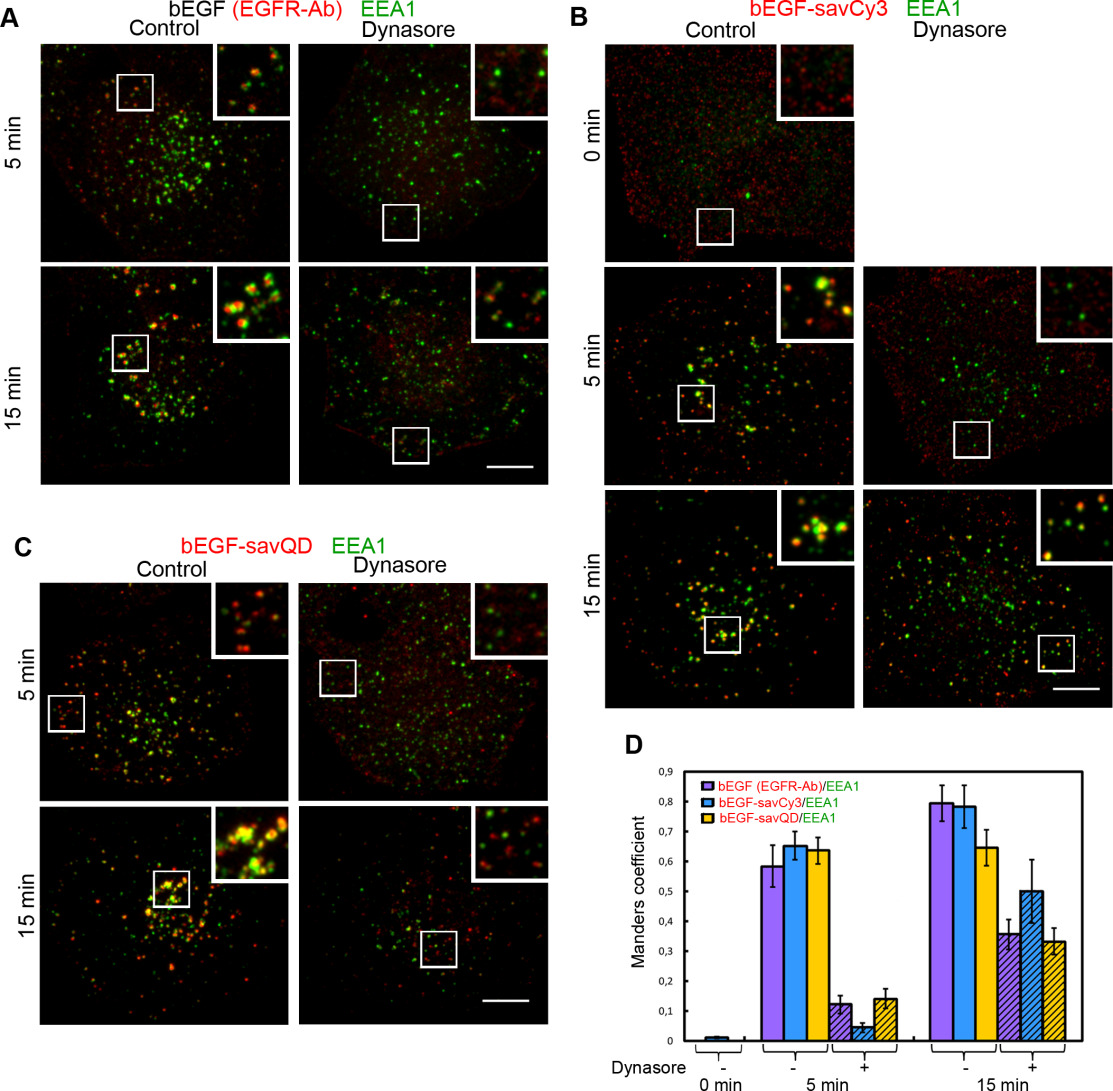
Immuno-co-localization of EGFR, bEGF-savCy3 and bEGF-savQD with EEA1 Control or dynasore-treated (80 μM) HeLa cells were incubated with (**A**) 2 nM bEGF, (**B**) 2 nM bEGF-savCy3 or (**C**) bEGF-savQD (2:0.5 nM) using prebinding (0 min) or pulse-chase (5 and 15 min) protocols and fixed. Then, for A, the cells were immunostained with anti-EGFR (Alexa 568) and anti-EEA1 (Alexa 488) antibodies before confocal microscopy. For (B and C), the cells were immunostained with anti-EEA1 (Alexa 488) antibody. (**D**) Co-localizations between EGFR-Ab, bEGF-savCy3 or bEGF-savQD and EEA1 in control (open columns) and dynasore-treated (shaded columns) cells were quantified using the Manders' coefficient (M1: red pixels overlapping green). Data presented as the mean ± 95% confidence interval of three independent experiments. Each image is representative of at least three independent experiments. The images are projections of Z-series onto single images using max intensity method (ImageJ). Insets represent enlarged views (2.2 ×) of boxed region. Scale bars: 10 μm.

According to established views, soon after endosome detachment, the EEA1 tether protein becomes associated with its membrane, allowing the process of early endosomal homotypic fusions to start [37]. In contrast to clathrin, no co-localization of bEGF-savCy3 and EEA1 was detected at the prebinding step (Figure 7B and 7D, 0 min), and the same was previously shown by us for EGF [38]. At 5 and 15 min of incubation, bEGF, bEGF-savCy3 and bEGF-savQD demonstrated equally high co-localization with EEA1; however, it was increased by 15 min for the first 2 ligands, except bEGF-savQD (Figure 7D). Interestingly, dynasore treatment practically blocked EEA1 association with EGFR-containing structures 5 min after ligand addition, but EEA1 interaction with them increased about 2–3 fold at 15 min (Figure 7). Also, in the presence of dynasore, co-localization of bEGF-savCy3 at 5 min was lower and at 15 min was higher than for two other ligands, but the reasons for this are not clear (Figure 7D). Nevertheless, in dynasore-treated cells, EEA1-positive structures were localized at the cell periphery while in the control they become translocated more juxtanuclearly (Figure 7A–7C). Additionally, dynasore prevented significant enlargement of vesicles in all cases. These data indicate that EEA1 can be recruited not only onto separated endosomes but also to PM-associated CCP structures, possibly partially uncoated but unable to fuse with each other.

**Figure 8:**
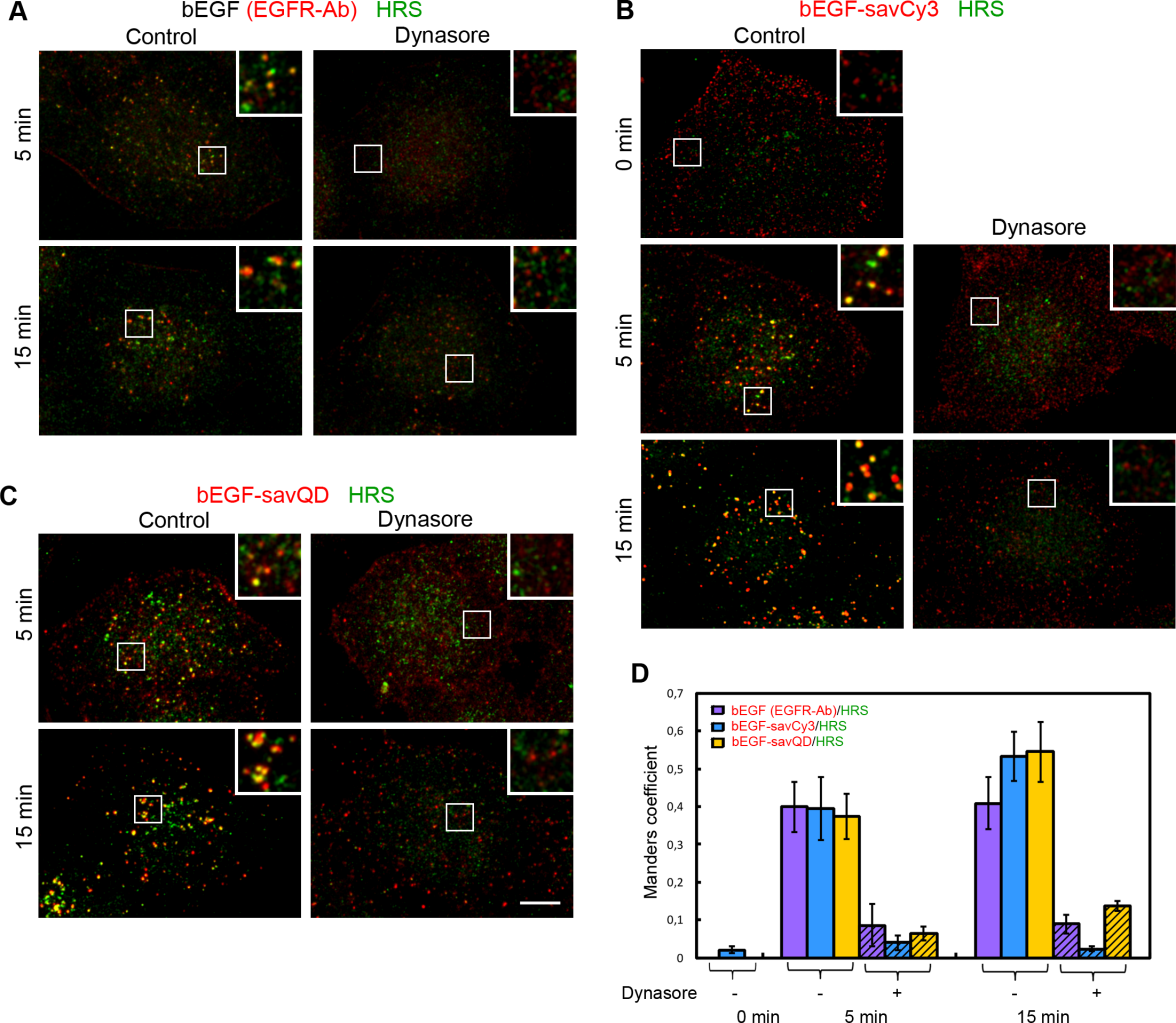
Immuno-co-localization of EGFR, bEGF-savCy3 and bEGF-savQD with HRS Control or dynasore-treated (80 μM) HeLa cells were incubated with (**A**) 2 nM bEGF, (**B**) 2 nM bEGF-savCy3 or (**C**) bEGF-savQD (2:0.5 nM) using prebinding (0 min) or pulse-chase (5 and 15 min) protocols and fixed. Then, for A, the cells were immunostained with anti-EGFR (Alexa 568) and anti-HRS (Alexa 488) antibodies before confocal microscopy. For (B and C), the cells were immunostained with anti-HRS antibody (Alexa 488) before confocal microscopy. (**D**) Co-localizations between EGFR-Ab, bEGF-savCy3 or bEGF-savQD and HRS in control (open columns) and dynasore-treated (shaded columns) the cells were quantified using the Manders' coefficient (M1: red pixels overlapping green). Data presented as the mean ± 95% confidence interval of three independent experiments. Each image is representative of at least three independent experiments. The images are projections of Z-series onto single images using max intensity method (ImageJ). Insets represent enlarged views (2.5 ×) of boxed region. Scale bars: 10 μm.

Furthermore, we have analyzed the co-localization of ligand-receptor complexes with HRS, the component of ESCRT0 complex, the first complex that meets EGFR to sort it to the degradative pathway. No co-localization was found for pre-bound bEGF-savCy3 at 4°C (Figure 8B and 8D, 0 min), but a significant portion of the label was associated with this marker at 5 min, showing a further slight increase by 15 min for the two quasi-multivalent ligands (bEGF-savCy3 and bEGF-savQD). In all cases, small yellow structures seen at 5 min were converted by 15 min into enlarged multidomain organelles or organelles clusters localized juxtanuclearly (Figure 8A–8C). Importantly, dynasore has drastically reduced co-localization for all ligands at both 5 and 15 min, thus indicating a HRS association with exceptionally intracellular structures. The existence of co-localization (M1 = 0.4) at 5 min, similar for all three studied ligands, may indicate that populations of bEGF-savQD complexes which have already become internalized at 5 min are equivalent with respect to the recognition by the sorting machinery, despite the differences in their initial steps of interactions with the cell.

## DISCUSSION

The implementation of QD as a label, in the case of targeting molecules such as the small EGF peptide undergoing internalization, could obviously have two shortcomings: a large size of functionalized QD and quasi-multivalency due to multiple EGF-binding sites on a QD particle. For such an enlarged ligand, the size of EGF-specific portal, CCP, which has a diameter of about 120–150 nm in mammalian epithelial cells [6, 20, 21], can be a limiting factor for proper packing in coated vesicles and further formation of primary endosomes. It also could be expected that QD implementation would produce essential changes in the dynamics of early endocytic events the correctness of which depends on the receptor dimerization and activation stimulated by ligand binding. However, there are very few detailed studies of the influence of the abovementioned factors on the biological relevance of QD-labeled EGF in respect of dynamics of early endocytic stages [10, 39].

According to the aims of our study, we first tested the way by which bEGF-savQDs enter HeLa cells. No differences between native EGF behavior described earlier [40, 41] and that of bEGF-savQDs was found: QD-labeled EGF enters the cells via clathrin- and dynamin-dependent, temperature-sensitive, EGFR-specific pathway (Figure 1). We also show that two other epithelial cell lines, A431 and PAE A11, internalize bEGF-savQD also through the EGFR-mediated mechanism (Supplementary Figure 1).

One of the most important factors is quasi-multivalency of bEGF-savQDs. As mentioned earlier, native EGF binds its receptor at a ratio of 1:1 [22], but it was established that dimerization of EGF-receptor complexes is strongly required for effective internalization and TK activation [23–25]. It was also demonstrated that EGF receptors may exist on the plasma membrane both in monomeric and inactive dimeric forms, and the ratio of the two populations is proportional to the number of surface receptors [42]. Obviously, EGF molecules bound to any artificial carriers need to be able to match the distance between two receptors in a dimer, which, according to the existing estimations, may range from 1.5 to11 nm [43, 44], depending on a certain conformation. In our case EGF bounds to QD through streptavidin-biotin system, where each QD particle contains several streptavidins and streptavidin *per se* is a homotetramer with no co-operativity in biotin molecules binding [45]. Therefore, practically all versions of streptavidin tetramers can be found on the same QD particle, from unligated ones to those associated with 1–4 bEGF molecules, with distances of about 2–3.5 nm for bound biotins [46]. These distances are of the same order of magnitude as in the case of receptor binding sizes. So it is expected that at least some of QD-localized bEGFs can successfully form proper complexes with EGF receptors.

Taking this into account, it can be supposed that the more bEGF binds a QD, the more the probability that bEGF-savQD will form a ligand-receptor dimer. To check this assumption we have analyzed how the number of bound bEGF per a QD particle will affect the number and intergral intensity of endosomes formed during 15 min of internalization of bEGF-savQD complexes prepared at variable concentrations of bEGF and savQDs. In HeLa cells bEGF-to-savQD concentration ratios ranging from 4:1 to 12:1, in contrast to 1:1 ratio, were “endocytotically efficient” and resulted in the formation of about 100 endosomes per cell on average 15 min after the ligand addition (Figure 4A and 4C). Additionally, very efficient competition of comparable concentration of free EGF with bEGF-savQD and the inhibiting effect of high (24:1) ratio used in the complex preparation (Figure 4A and 4C) as well as the observed non-uniform receptor content in bEGF-savQD-positive structures (Figure 1B) indicate that QD-labeled EGF can form numerous configurations of ligand-receptor complexes. Most of these configurations are binding-defective. Thus, it must be taken into consideration that nominal bEGF concentration even in the case of optimal ratios actually means a significantly lower acting bEGF concentration.

Importantly, in our study bEGF-savQD complexes formed at a 1:1 ratio were “endocytotically inefficient” in HeLa cells: after 15 min pulse very few endosomes with low integral intensity could be found. However, 1:1 complexes were internalized successfully by A431 cells overexpressing EGFR (Figure 4B and 4E). This ratio seems attractive for single molecule labeling, and was previously reported to be endocytotically active [7]; however, the cited work was also fulfilled on A431 cells. Overexpression of EGFR usually results in high receptor density on the PM and is known to correlate with high level of ligand-independent internalization [13, 14, 47]. We suppose that the ability of bEGF-savQD complexes prepared at 1:1 ratio to enter the cells can be used as a marker for cells with very high receptor density typical of many transformed cells. However, for the cell lines expressing medium receptor number (about 10^5^ molecules per cell) with a low level of basal receptor internalization only endocytotically effective ratios provide a high probability of receptor dimerization and subsequent internalization.

Light microscopy limitations makes it difficult to differentiate between QD entrapped in coated pit and in fully detached vesicle just near the surface. Moreover, our data presented in the video (see Supplementary Video) in combination with our experiments on endocytosis inhibition by dynasore (Figure 5), which prevents the detachment of CCPs [32], suggest that bEGF-savQDs can hardly be detected in CCP in the absence of the drug due to a low degree of the label concentration there. Dynasore treatment data also support the idea that the bright, reliably detected structures at early stages are endosomes (Figure 5). We quite expectedly found that in the control cells the brightness of the labeled structures increased with time, which reflects cargo concentration during endosomes formation and the following fusions. However, in the presence of dynasore this increase was significantly slower. This indicates that though some concentration of the label occurs in coated pits, the main sites of cargo concentration are fusing endosomes. These data are in agreement with the dynamics of native EGFR endocytosis described earlier elsewhere [26, 30].

However, some differences were revealed between bEGF-savQD, a ligand of bigger size, and the smaller bEGF-savCy3 in the dynamics of their interaction with the cell surface at early stages. First, the two ligands have different preferable areas for binding (Figure 2). If the exclusion of QDs from the basal domain of PM can be explained by size limitations of bEGF-savQD penetration into a narrow space full of extracellular matrix between the cell bottom and coverslip, the reasons for different distributions of bEGF-savCy3 and bEGF-savQD onto the rest of the PM are not clear. Second, the QD-labeled EGF binds the receptors and enters CCPs slower than bEGF-savCy3 which behaves similarly to native EGF. This conclusion is supported by experiments showing that acidic washout efficiently dissociates the ligand-receptor complexes localized outside coated pits (Figure 5), because it was established [48, 49] that entrapment into coated pits makes EGF-EGFR complexes more stable, possibly due to additional interaction with CCP components. In our study the QD-labeled ligand turns to be more sensitive to this washout than bEGF-savCy3. By our estimations about 25% of bEGF-savQD-receptor complexes stay out of coated pits even at 15 min (Figure 5D). Also, the comparison of brightness dynamics for Cy3- and QD-labeled endosomes in dynasore-treated cells that can accumulate the ligand only in CCPs has revealed a slower accumulation rate for bEGF-savQD-positive structures (Figure 5B and 5D). Yet, though these differences are statistically reliable, they are not significant.

Our data also demonstrate that clathrin is not a good marker for solving the problem of differentiation between CCPs and already detached vesicles for two reasons. First, the amount of both the free and membrane-bound clathrin in HeLa cells is very high, providing sufficient background signals. Second, clathrin lattices were reported to be associated not only with PM and Golgi, but also with early endosomal membranes [34–36]. In our experiments co-localization of EGFR, as well as of bEGF-savCy3, with clathrin was comparable or even higher in the cells allowed to internalize ligand-receptor complexes for 15 min than in the cells with inhibited internalization (Figure 6). This result suggests that uncoated early endosomal vesicles become again associated with clathrin several minutes after their detachment from PM.

Association of receptor complexes, formed by all three ligands, with a widely used early endosome marker tether protein EEA1 was found to be similar. Interestingly, though this process was strongly inhibited by dynasore at 5 min pulse, we have found that dynasore does not prevent the accumulation of EEA1 on receptor-containing structures by 15 min (Figure 7). This may reflect a previously unknown ability of this protein, which mediates the first stage of homotypic endosome fusion, to recognize undetached vesicles, possibly partially uncoated. However, we can not also exclude the possibility of incomplete inhibition of endocytosis by dynasore during a relatively long incubation.

We have demonstrated that in control cells co-localization of all 3 ligands and HRS even at 5 min was relatively high and it was completely inhibited by dynasore (Figure 8). The dynamics of HRS co-localization with the ligands tested was also similar in all 3 cases. Thus, only at the stage of HRS recruitment a reliable differentiation between internalized vesicles and those associated with PM is possible. Considering that internalization is controlled by the receptor dimerization rather than its TK activation, while the latter is necessary for both the association with HRS as a component of ESCRT0 complex and proper receptor signaling [23, 25, 41, 50], we can conclude that despite some slowing down of very early steps of QD-labeled EGF binding with the cells, a high degree of co-localization of bEGF-savQD and HRS at 15 min indicates that both the cargo sorting and endosomal maturation processes develop properly.

In conclusion, the data presented show that in comparison with the behavior of small size monovalent native EGF the implementation of large size quiasi-mulivalent fluorophore bEGF-savQD as a label causes insignificant differences only at the stage of binding to the surface EGF receptors and formation of dimers necessary for efficient internalization. Keeping the concentration ratio of bEGF to QD in the range of 4:1 – 12:1 significantly increases the probability of the dimer formation and consequently provides the normal process of packing the cargo into the forming vesicles and their detachment. Furthermore, the early stages of endosomes functioning, like homotypic fusions and sorting of the cargo to degradative pathway by ESCRT complexes are similar for all 3 ligands tested. Thus, QD can be used as a perfect tool to study many aspects of EGFR-dependent intracellular processes in basic research. Due to the high specificity of QD-labeled EGF interaction with the cells and demonstrated here preferable accumulation of the label in the cells overexpressing EGF receptors, it can be used for target delivery of multifunctional therapeutic drugs in case of cancers with high level of EGF receptors. However, it can be supposed that QD-labeled EGF is able to form receptor complexes with variable signaling efficacy. It means that the receptor activation may be different in differently organized receptor complexes and there are numerous data on the significance of the EGFR activation pattern for endocytic pathway and signaling outcome (reviewed in [51]). Therefore, direct studies of this problem are necessary for the final assesment of the biological relevancy of QD implementation.

## MATERIALS AND METHODS

### Reagents and antibodies

Epidermal growth factor biotin conjugate (bEGF), CdSe/ZnS Qdot streptavidin conjugate with emission maximum at 655 nm (savQD) and Cy3-streptavidin (savCy3) were purchased from Invitrogen (USA). Mouse native EGF, dynasore and Hoechst 33258 were from Sigma-Aldrich (USA). Rabbit polyclonal anti-EGFR antibody (#2232) was from Cell Signaling Technology (USA), mouse monoclonal anti-EEA1 antibody (#610457) was from BD Transduction Lab (USA), mouse monoclonal anti-clathrin heavy chain antibody X22 (#CP45) was from Merck Millipore (Germany) and mouse monoclonal anti-HRS antibody (#ALX-804-382-C050) was from EnzoLifeSciences (USA). Alexa Fluor 568 goat anti-rabbit IgG and Alexa Fluor 488 goat anti-mouse IgG were from Invitrogen (USA). Other chemicals were from Sigma-Aldrich unless otherwise stated.

### Cell culture

Human cervix epidermoid carcinoma HeLa cells and squamous carcinoma A431 cells (Russian Cell Culture Collection, Institute of Cytology RAS, St. Petersburg, Russia) were maintained in Dulbecco's modified Eagle medium (DMEM, Biolot, Russia) with 10% fetal bovine serum (FBS, Biolot, Russia) and 1% penicillin/streptomycin (GIBCO, USA) incubated at 37°C with 5% CO_2_. Porcine aortic endothelial (PAE) A11 cells stably expressing EGF-receptor-GFP (EGFR-GFP) (a kind gift of Dr. Sorkin, University of Pittsburgh School of Medicine, Pittsburgh, USA) were grown in DMEM/F12 containing 10% fetal bovine serum, antibiotics and 500 ng/ml geneticin (G418) (Mediatechnic, USA). Cells were seeded in Lab-Tek borosilicate coverglass-bottomed chambered slides (Nunc) for live cell imaging or on Petri dishes with glass coverslips (Nunc). Cells were starved (0.1% FBS) overnight. Experiments were held at 60–70% confluent, 48 h after seeding.

### Ligand preparation

bEGF-savCy3 or bEGF-savQD complexes were prepared *in vitro* in PBS at 4°C by mixing for 30 min. The bEGF-savCy3 complex was prepared using 2 nM of bEGF and 30-fold excess of savCy3 considering to obtain 1:1 ratio of the bEGF to savCy3. To prepare the bEGF-savQDs complex used in most experiments, 2 nM of bEGF was mixed with 0.5 nM of savQDs. To study the influence of variable bEGF to savQD ratios on endocytosis, concentrations were used as indicated in Figure 4. Free EGF or bEGF was added at the concentrations indicated in corresponding figure legends.

### Stimulation of endocytosis

The pulse-chase protocol was chosen to stimulate endocytosis under physiological conditions. Cells were washed twice with warm (37°C) DMEM and pulsed for 5 min with bEGF, bEGF-savCy3 or bEGF-savQD at 37°C. Then, the unbound ligands were intensively washed out with warm DMEM and the cells were fixed (this point is further designated as 5 min pulse) or chased for an additional 10 min at 37°C before fixation (15 min pulse).

To analyze EGF interaction with only the surface receptors in the absence of internalization, we used the prebinding protocol. Where indicated, the cells were washed twice with cold (4°C) DMEM, placed on ice and bEGF (2 nM) in DMEM was added for 40 min. Unbound ligand was then washed out by 3 rinses with cold DMEM. Then, cells were incubated on ice for 40 min with savCy3 or savQD, washed and fixed.

### Incubation under inhibitory conditions

Hypertonic sucrose solution (0.45 M) preventing normal clathrin lattice assembly was used to inhibit clathrin-dependent endocytosis and dynasore (80 μM) was used to inhibit GTP-ase activity of dynamin and prevent the separation of coated pits from the PM. For this, the cells were incubated for 30 min with a drug. Afterward, the cells were exposed to a solution containing the same drug concentration plus bEGF, bEGF-savCy3 or bEGF-savQDs for the indicated time.

### Acidic washout

After incubation with bEGF-savCy3 or bEGF-savQD for the indicated time, the cells were placed on ice and washed with cold acetic buffer (0.2 M acetic acid/0.5 M NaCl, pH 4.0) 3 times for 2 min to remove surface-bound EGF, followed by 4 rinses with DMEM. Then, the cells were fixed for imaging and analysis.

### Immunofluorescent staining

The cells were fixed with 4% paraformaldehyde for 15 min, permeabilized with 0.5% Triton X-100 for 15 min and blocked with 1% BSA for 1 h. Fluorescence of bEGF-Cy3 and bEGF-QD was detected directly. To reveal EGFR localization the fixed cells were incubated with primary anti-EGFR antibody (1:100) for 24 h at 4°C and then for 1 h with Alexa 568 or 488 goat anti-rabbit IgG (1:500). For co-localization analysis the cells were additionally incubated for 1 h at room temperature with primary antibodies of choice (anti-clathrin heavy chain antibody in 1:2000 or anti-HRS in 1:500 dilutions, anti-EEA1 antibody at 0.25 μg/ml concentration) and for 1 h with secondary antibodies (Alexa 488 goat anti-mouse IgG, 1:500). After immunostaining the cells were mounted into Fluorescent Mounting Medium (Dako Cytomation, Denmark).

### Confocal microscopy

The cells were examined with Leica TCS SP5 inverted laser scanning confocal microscope (Germany) equipped with solid-state lasers for excitation (405, 488 and 543 nm). QD fluorescence was excited at 405 or 488 nm and registered in the 640–670 nm channel; Cy-3 fluorescence was excited at 543 nm and registered in the 560–620 nm range. Alexa 488 and Alexa 568 were excited at 488 nm and 543 nm and registered in the 500–550 and 580–660 nm ranges, respectively. GFP was excited at 488 nm and registered in the 500–550 nm range. Hoechst fluorescence was excited at 405 nm and registered in the 430–480 nm range. Specimens were observed with a × 40 oil immersion objective, followed by a 4 digital zoom magnification with an image size of 1024 × 1024 pixels. Images were taken in one or two spectral channels by sequential scanning mode, where only one laser was active at a time, to avoid spectral overlap. To optimize the signal to noise ratio, the final image was an average of three consequent runs. Z-series optical sections were taken at 0.5-μm intervals from the bottom to the top (14–16 sections). Images were acquired for at least 5 fields of view selected randomly per coverslip. Data were collected by Leica software as raw *.lif files and transferred as a series of tiff files for further analysis.

### Live cell imaging

The cells seeded in Lab-Tek chambers and incubated with bEGF-savQD at indicated conditions were analyzed with Leica TCS SP5 confocal microscope equipped with temperature and gas control chamber (37°C and 5% CO_2_). Specimens were observed with a × 40 oil immersion objective, followed by a 4 digital zoom magnification with image size of 1024 × 1024 pixels. Simultaneous confocal images of QD and brightfield images of cells were obtained. For the vital staining of nuclei, Hoechst 33258 was used at a concentration of 1.6 μM for 5 min. For time-lapse video, PAE A11 cells were imaged at 1 frame per 5 sec for an 11 min period (148 images). The movie was compressed to 10 frames per second.

### Image and statistical analysis

All data were obtained from at least three independent experiments. In each experiment, 4–5 fields containing 15–20 cells totally were imaged for each time point. The images were processed and analyzed using Leica Confocal Software (Germany) and ImageJ software (National Institute of Health). In confocal images, the background of each channel was subtracted and, in some cases, brightness/contrast was adjusted only for presentation. No filter was applied in quantitative analyses. Z-stacks were projected onto single images (projections) using the max intensity method (ImageJ). Then, single sections or projections were exported to Adobe Photoshop 5.0 for final image processing.

Analysis of Region of Interest (ROI) intensities was carried out using ImageJ. The number and integrated intensities of endosomes were measured from max intensity projections using ImageJ (menu command Analyze). The quantitative co-localization analysis was performed using ImageJ JACoP Plugin [52] to determine Manders' coefficient (M1: red pixels overlapping green pixels). Thresholds were set by a visually estimated value for each channel.

For all quantitative analyses, the results are presented as the mean ± 95% confidence interval for at least fifteen cells. The column charts were created using Microsoft Office Excel 2007.

## SUPPLEMENTARY MATERIALS FIGURES AND VIDEO





## Abbreviations

QDs: Quantum dots
EGFR: epidermal growth factor receptor
b: biotin
sav: streptavidin
EEA1: early endosome antigen 1
ESCRT: endosomal sorting complex required for traffic
HRS: hepatocyte growth factor-regulated tyrosine kinase substrate
TK: tyrosine kinase
PEG: polyethylene glycol
CCP: clathrin-coated pit
EE: early endosome
PM: plasma membrane
