# Supplementary material for: Quantum dots implementation as a label for analysis of early stages of EGF receptor endocytosis: a comparative study on cultured cells
Source: Oncotarget. 2015 Dec 22;7(5):6029–47. doi: 10.18632/oncotarget.6720 (PMC4868738; doi:10.18632/oncotarget.6720)
Supplement: Supplementary file 1 [file oncotarget-07-6029-s001.pdf]

Quantum dots implementation as a label for analysis of early stages of EGF receptor endocytosis: a comparative study on cultured cells

Supplementary Materials

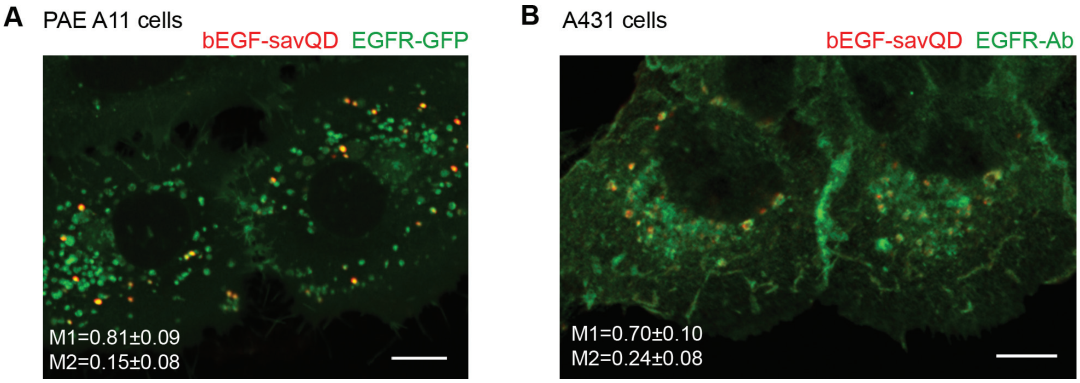

Supplementary Figure S1: Specificity of bEGF-savQD entry into the cells. (**A**) PAE A11 cells expressing EGF-receptor-GFP were incubated with bEGF-savQD (2:0.5 nM) for 15 min at 37°C before live confocal microscopy. (**B**) A431 cells were incubated with bEGF-savQD (2:0.5 nM) for 15 min at 37°C, fixed and immunostained with anti-EGFR antibody (Alexa 488) before confocal microscopy. Each image was taken as a single section from the region of maximal cell spreading and is representative of at least three independent experiments. Scale bars: 10 µm. The Manders' coefficients of bEGF-savQD and EGFR co-localization (M1 – red pixels overlapping green, and M2 – green pixels overlapping red) indicate that practically all QD-labeled EGF is co-localized with the receptor, while the majority of receptors due to their high expression level are not.

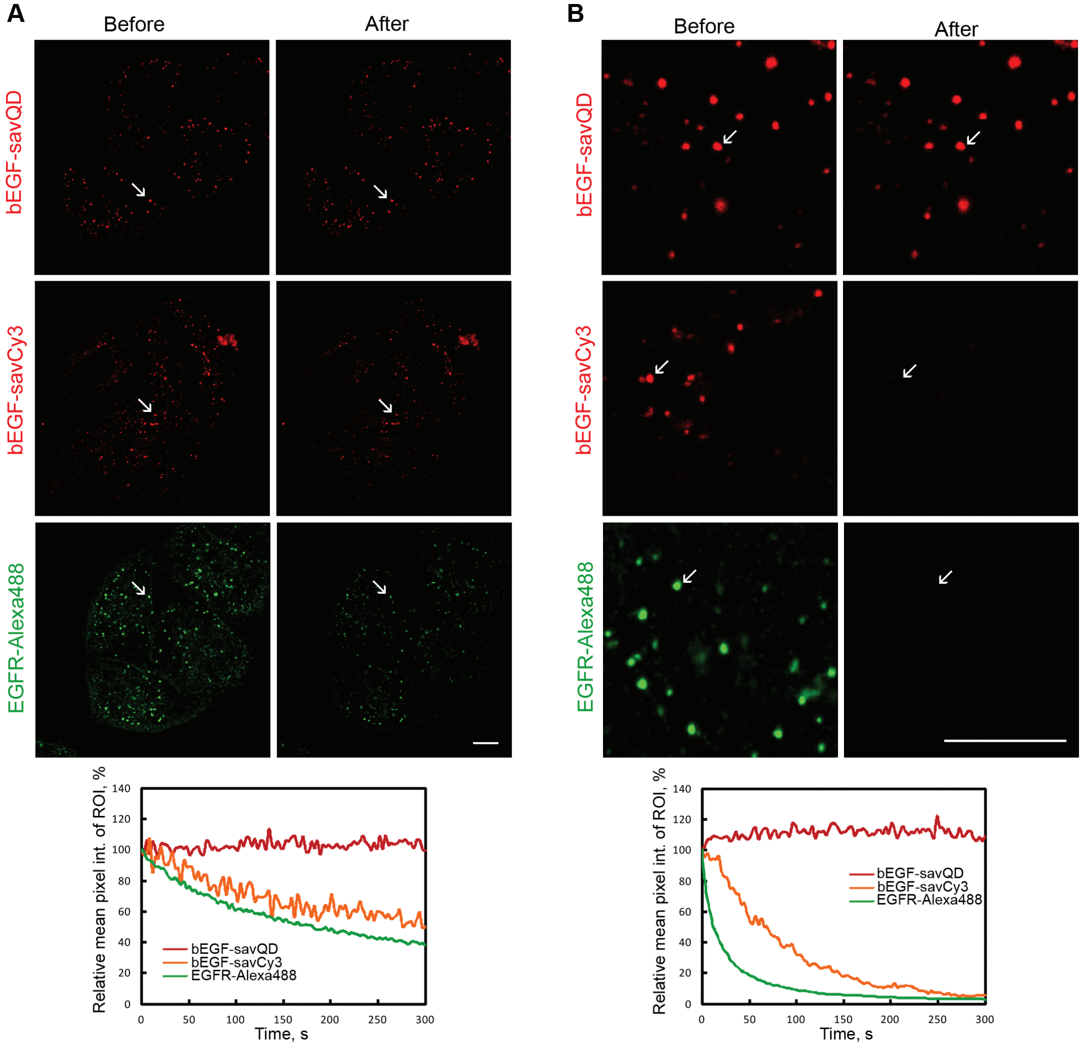

Supplementary Figure S2: Photostability of fluorescent markers for endocytosis investigation. HeLa cells were incubated (pulse-chased) with bEGF-savQD, bEGF-savCy3 or native EGF for 15 min at 37°C, washed out of unbound ligands, fixed and, in the case of native EGF, immunostained with anti-EGFR (Alexa 488) antibody. For each case, representative temporal series of 101 images (every 3 sec during 5 min) were taken using a confocal fluorescence microscope Leica TCS SP5. Initial and final images of the series are presented. Scale bars: 10 µm. The laser power was the same in (**A**) and (**B**), but the size of the illuminated area was 97 × 97 µm (A) and 19.4 × 19.4 µm (B). The plots represent time dependence of relative mean pixel intensity of the Region of Interest (ROI) which corresponds to one endosome indicated by an arrow for each fluorophore. The intensity behavior of at least 20 structures was similar for each fluorophore. Evidently more intensive illumination in (B) resulted in a more drastic decrease in fluorescence of the both Cy3- and Alexa488-labeled EGF than in (A). Importantly, the intensity of QD signals practically did not change in either case. The plots are representative for three independent experiments.

Supplementary Video: The early stages of bEGF-savQD interaction with PAE A11 cells, expressing GFP-labeled EGFR. A representative time-lapse video demonstrates the process of QD interaction at the edge of the highly spreaded cell during the first 11 min after the addition of bEGF-savQD (2-0.5 nM) complexes to the cells at 37°C. Red channel (640–670 nm) represents QD fluorescence, while the fluorescence of EGFR-GFP was registered in green channel (500–550 nm). Here, 148 frames were taken every 5 sec. The movie was compressed to 10 frames per second. Note the highly dynamic meshwork of dimmed fluorescent dots appearing and disappearing in areas close to the cell edge and emerging bright structures indicated by arrows. Scale bar: 1 µm.
